# Similar Efficacy of Arthroscopy and Arthrotomy in Infection Eradication in the Treatment of Septic Knee: A Systematic Review and Meta-Analysis

**DOI:** 10.3389/fsurg.2021.801911

**Published:** 2022-01-13

**Authors:** Zhimin Liang, Xiaofan Deng, Lingli Li, Jing Wang

**Affiliations:** ^1^School of Nursing, West China Hospital, Sichuan University, Chengdu, China; ^2^Department of Orthopaedic Surgery, West China Hospital, Sichuan University, Chengdu, China; ^3^Organ Transplant Center, Sichuan Provincial People's Hospital, Sichuan Academy of Medical Sciences, Chengdu, China

**Keywords:** septic arthritis, knee surgery, arthroscopy, arthrotomy, systematic review and meta-analysis (Level III)

## Abstract

**Aim:** To compare the arthroscopy vs. arthrotomy for the treatment of native knee septic arthritis.

**Methods:** Electronic databases of PubMed, Embase and Cochrane Library were searched for eligible studies. Retrospective comparative studies comparing arthroscopy or arthrotomy for patients with septic arthritis of the native knee were eligible for this review. The primary outcome was recurrence of infection after first procedure. The secondary outcomes included hospital length of stay, operative time, range of motion of the involved knee after surgery, overall complications and mortality rate,

**Results:** Thirteen trials were included in this study. There were a total of 2,162 septic arthritis knees treated with arthroscopic debridement and irrigation, and 1,889 septic arthritis knees treated with open debridement and irrigation. Arthroscopy and arthrotomy management of the knee septic arthritis showed comparable rate of reinfection (OR = 0.85; 95% CI, 0.57–1.27; *P* = 0.44). No significant difference was observed in hospital length of stay, operative time and mortality rate between arthroscopy and arthrotomy management group, while arthroscopy treatment was associated with significantly higher knee range of motion and lower complication rate when compared with arthrotomy treatment.

**Conclusion:** Arthroscopy and arthrotomy showed similar efficacy in infection eradication in the treatment of native septic knee. However, arthroscopy treatment was associated with better postoperative functional recovery and lower complication rate.

## Introduction

Septic arthritis is a serious orthopedic emergency that can lead to devastating cartilage destruction and even be life threatening. The incidence of septic arthritis is 4–10 per 100,000 people per year in western countries and the frequency is reported to increase (1–4). The most common joint affected is knee, which is involved in about half of the septic arthritis cases (4).

The diagnosis of septic arthritis relies on clinical symptoms, laboratorial exams, elevated inflammatory markers in synovial fluid, positive culture of the joint fluid and histopathological examination. Immediate management is essential to prevent devastating cartilage destruction and sepsis. Early diagnosis and treatment determine the final outcome, and it has been reported that the success rate of treatment was associated with the time from diagnosis to treatment initiation (5). The principle of treatment is intravenous antibiotics combined with emergency surgery to decrease the intra-articular microbial burden (6, 7). As repeated needle aspiration alone has been proved to be insufficient to eradicate the infection and should only be performed at very early stage (8, 9), arthroscopy and arthrotomy with thorough debridement and irrigation have been discussed recently and both showed reliable results (10).

A number of trials comparing arthroscopy vs. arthrotomy in the treatment of knee septic arthritis has been published. However, controversial results exist regarding the ideal approach to the knee septic arthritis. Some trials indicated arthroscopy and arthrotomy had comparable postoperative outcomes (11, 12), while the results of some studies favored arthroscopy (13, 14). A recently published meta-analysis in this field included seven studies with 1,089 knees, and concluded that arthroscopy treatment could result in lower re-operation rate and better functional outcome than arthrotomy (15). Several additional trials comparing arthroscopy and arthrotomy have been published in recent years, which merits an updated meta-analysis.

Therefore, the purpose of this study is to compare the efficacy of arthroscopy and arthrotomy management in the treatment of septic arthritis of the native knee. The efficacy of infection eradication, operative parameters, postoperative functional recovery and complications after arthroscopy and arthrotomy treatment were investigated and compared in this systematic review and meta-analysis.

## Materials and Methods

This study has been preregistered in PROSPERO (ID: CRD42019146663). This manuscript was conducted according to the PRISMA (Preferred Reporting Items for Systematic Reviews and Meta-Analyses) guidelines (16).

### Search Strategy

Electronic databases of PubMed, Embase and Cochrane Library were searched for eligible studies in June 2021. There was no language restriction and no time frame was specified as for date of publication. The following keywords with relevant Boolean operator were used: Septic Arthritis, Arthroscopy, Arthrotomy, Open Management and Knee. The search strategy details have been shown in Table 1. Manual searching for additional eligible studies was also performed.

**Table 1:** Search strategy.

**Database**	**Search strategy**
Pubmed	(Septic Arthritis[tiab] OR Suppurative Arthritis[tiab] OR infect* Arthritis[tiab] OR Pyogenic Arthritis[tiab] OR Bacterial Arthritis[tiab] OR Arthritis, Infectious[MeSH]) AND (Arthrotomy[tiab] OR Open[tiab] OR Arthroscop*[tiab] OR Arthroscopy[MeSH]) AND (Knee*[tiab] OR Knee Joint[MeSH] OR Knee[MeSH])
Cochrane library	#1 Septic Arthritis OR Suppurative Arthritis OR infect* Arthritis OR Pyogenic Arthritis OR Bacterial Arthritis:ti,ab,kw #2 MeSH descriptor: [Arthritis, Infectious] explode all trees #3 Arthroscop* OR Arthrotomy OR Open:ti,ab,kw #4 MeSH descriptor: [Arthroscopy] explode all trees #5 Knee*:ti,ab,kw #6 MeSH descriptor: [Knee] explode all trees #7 MeSH descriptor: [Knee Joint] explode all trees #8 (#1 OR #2) #9 (#3 OR #4) #10 (#5 OR #6 OR #7) #11 (#8 AND #9 AND #10)
Embase	#1 'Septic Arthritis':ab,ti OR 'Suppurative Arthritis':ab,ti OR 'infect* Arthritis':ab,ti OR 'Pyogenic Arthritis':ab,ti OR 'Bacterial Arthritis':ab,ti #2 'infectious arthritis'/exp #3 'Arthroscop*':ab,ti OR 'Arthrotomy':ab,ti OR 'Open':ab,ti #4 'arthroscopy'/exp #5 'Knee*':ab,ti #6 'knee'/exp #7 (#1 OR #2) #8 (#3 OR #4) #9 (#5 OR #6) #10 (#7 AND #8 AND #9)

### Article Selection

Eligible studies were retrospective comparative studies comparing arthroscopy and arthrotomy management for patients with septic arthritis of the native knee. Studies were excluded if they included patients with any implant in the septic knee, for example, patients suffering from periprosthetic infection after total knee arthroplasty, or infection after implanting internal fixation hardware, or infection after ligament reconstruction using grafts. Two independent reviewers assessed the titles and abstracts for initial screening. Disagreements were resolve by discussion and consensus. When the decision was still not reached, a third reviewer's opinion was sought. Articles selected from initial screening underwent full-text review.

### Study Outcome and Data Extraction

The primary outcome measure of interest was recurrence of infection after first procedure, which needed to return to operation room for a second procedure. The secondary outcomes included hospital length of stay, operative time, range of motion of the involved knee after surgery, overall complications and mortality rate. Patient demographics, details of septic arthritis and follow-up time were extracted from included studies.

### Assessment of Risk of Bias

The risk of bias of retrospective comparative studies was evaluated according to Risk Of Bias In Non-randomized Studies-of Interventions tool (ROBINS-I), which was categorized as low risk, moderate risk, high risk, critical risk of bias or no information on the basis of bias due to confounding, bias in selection of participants, bias in measurement of interventions, bias due to departures from intended interventions, bias due to missing data, bias in measurement of outcomes and bias in selection of reported results (17). The risk of bias was assessed by 2 independent reviewers.

### Statistical Analysis

Odds Ratio (OR) and 95% confidence intervals (CI) were calculated for dichotomous outcomes using Mantel-Haenszel (M-H) method. Mean difference (MD) and 95% CI were calculated for continuous outcomes using the Inverse Variance (IV) method. A random effects model was used to analyze the primary and secondary outcomes. We calculated the mean and SD according to Hozo et al. for outcomes without mean ± standard deviation (SD) (18). *I*^2^ > 50% represents significant heterogeneity in this review (19). *P* < 0.05 was defined as significance. Review Manager 5.2 software were used for statistical analyses.

## Results

### Article Selection

**Figure 1:**
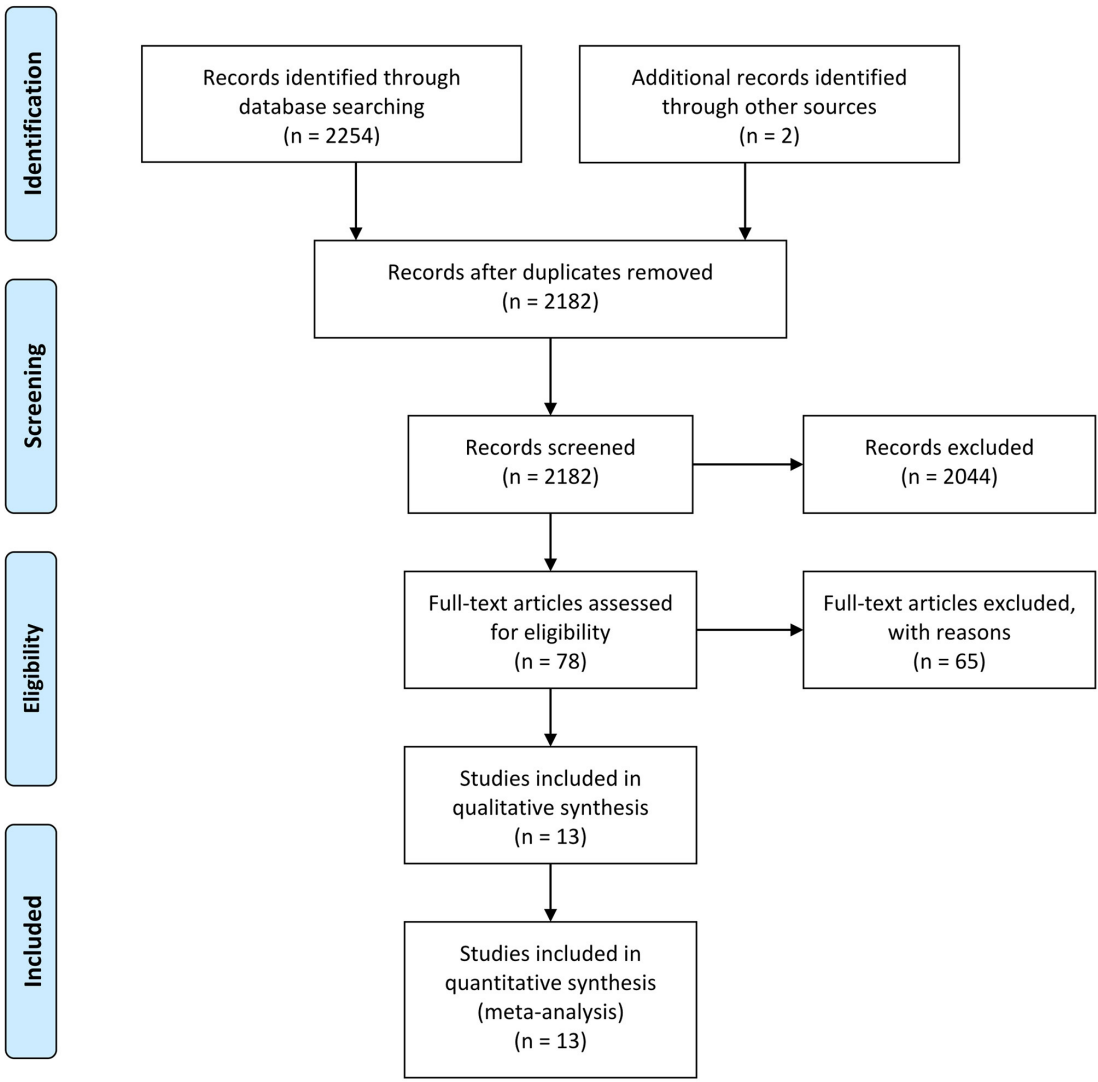
PRISMA flow diagram.

Initial trial search yielded 2,254 studies. After duplicates removed, a total of 2,182 studies underwent initial screening for the titles and abstracts. 78 studies selected from initial screening underwent full-text review and ultimately, 13 studies were included in this meta-analysis (11–14, 20–28). All included studies were retrospective cohort studies. The flow diagram of this review is presented in Figure 1.

We included thirteen retrospective comparative studies published from 2001 to 2021. A total of 4,051 native knees diagnosed as septic arthritis were enrolled in this meta-analysis (2,162 treated with arthroscopic debridement and irrigation and 1,889 treated with open debridement and irrigation). Details of the included studies were presented in Table 2.

**Table 2:** Characteristic of the included studies.

**References**	**Region**	**No. of patients**		**Male (%)**		**Age (yr)**		**Diagnosis**	**Culture**	**Primary outcome**	**Follow up**
		**Arthroscopy**	**Arthrotomy**	**Arthroscopy**	**Arthrotomy**	**Arthroscopy**	**Arthrotomy**				
Wirtz et al. (20)	Germany	27	24	25 (49.0%)		Mean 59.7 (range, 21–94)		Preoperative joint aspiration	Positive culture = 38 cases, most often SA Negative culture = 13	Reinfection within 2.2 years	Mean 2.2 years, max 12.8 years
Balabaud et al. (23)	France	21	19	31 (77.5%)		Mean 49 ± 20 (range 19–81)		Clinical symptoms, laboratory examination, preoperative joint aspiration	SA = 12 MRSA = 4 Negative culture = 3	/	Mean 22 ± 26 months, range 12–96 months
Werasak and Witchate (28)	Thailand	33	44	17 (51.5%)	21 (47.7%)	56.6 ± 16.6	58.6 ± 15.6	Clinical symptoms, laboratory examination	SA = 17	Operative time	NA
Böhler et al. (14)	Austria	41	29	27 (65.9%)	19 (65.5%)	Median 49 (Q1–Q3, 30–64)	Median 71 (Q1–Q3, 65–78)	Clinical symptoms, preoperative joint aspiration or histopathological examination	NA	Reinfection within 3 months	Median 12 months
Dave et al. (22)	United States	40	12	28 (53.8%)		Mean 43.4 (SD 23.8)		Clinical symptoms, laboratory examination, preoperative joint aspiration	SA = 15 MRSA = 5 Negative culture = 20	Reinfection	Mean 7.2 years, max 16/2 years
Jaffe et al. (11)	United States	33	47	22 (66.7%)	26 (55.3%)	Mean 59.7 (range, 54.5–64.9)	Mean 47.3 (range, 43.2–51.4)	Clinical symptoms, laboratory examination, preoperative joint aspiration	SA = 28 MRSA = 19 Negative culture = 22	Reinfection within 4 months	4 months
Bovonratwet et al. (12)	United States	216	168	56 (33.3%)	112 (66.7%)	Mean 60	Mean 58	ICD-9	NA	Return to the operating room	30 days
Johns et al. (13)	Australia	119	42	80 (67.2%)	28 (66.7%)	Mean 57.5	Mean 65.8	Clinical symptoms, laboratory examination, preoperative joint aspiration	Positive culture = 138 cases, most often SA Negative culture = 28	Reinfection	NA
Kalem and SAhIN (25)	Turkey	13	11	8 (61.5%)	6 (54.5%)	56.6 ± 14.9	59.5 ± 17.2	Clinical symptoms, laboratory examination, preoperative joint aspiration	Gram-positive bacteria = 4, MRSA = 2	Reinfection	6 months
Faour et al. (21)	United States	231	464	151 (65.3%)	302 (65.1%)	Mean 59 (SD 18)	Mean 59 (SD 15)	ICD-9	NA	Reinfection within 30 days	30 days
Johnson et al. (24)	United States	816	454	539 (66.1%)	310 (68.3%)	57.4 ± 17.9	57.3 ± 16	ICD-9 and ICD-10	NA	Operative time	30 days
Sabater-Martos et al. (27)	Spain	12	15	18 (66.7%)		64.8 (range 30–89)		Clinical symptoms, preoperative joint aspiration	SA = 8 Negative culture = 12	Reinfection	52.8 ± 11.2 months
Kerbel et al. (26)	United States	560	560	NA		412 (73.6%)	412 (73.6%)	ICD-9	NA	Major and minor complications	NA

*SA, staphylococcus aureus; MRSA, methicillin-resistant staphylococcus aureus; SD, standard deviation; NA, not available*.

### Assessment of Risk of Bias

Among the thirteen studies, nine had moderate risk of bias and four had high risk of bias (Table 3). All of the studies showed a moderate or serious confounding bias due to inadequate detail of age, stage of infection or culture. Bias in selection of participates were considered moderate in all studies because there may be an association between the interventions and outcomes (septic knee patients with mild and short-term symptoms were more likely to receive arthroscopic debridement and irrigation). Bias in measurement of interventions, bias due to departures from intended interventions and bias in measurement of outcomes were all judged low because the intervention (operation) and outcomes were objective and insusceptible.

**Table 3:** Risk-of-bias assessment of the retrospective cohort studies by ROBINS-I.

**References**	**Bias due to confounding**	**Bias in selection of participants**	**Bias in measurement of interventions**	**Bias due to departures from intended interventions**	**Bias due to missing data**	**Bias in measurement of outcomes**	**Bias in selection of reported results**	**Overall bias**
Wirtz et al. (20)	Serious	Low	Low	Low	Moderate	Low	Low	High risk of bias
Balabaud et al. (23)	Moderate	Low	Low	Low	Low	Low	Low	Moderate risk of bias
Werasak and Witchate (28)	Serious	Low	Low	Low	Moderate	Low	Low	High risk of bias
Böhler et al. (14)	Moderate	Low	Low	Low	Low	Low	Low	Moderate risk of bias
Dave et al. (22)	Moderate	Low	Low	Low	Low	Low	Low	Moderate risk of bias
Jaffe et al. (11)	Moderate	Low	Low	Low	Low	Low	Low	Moderate risk of bias
Bovonratwet et al. (12)	Moderate	Low	Low	Low	Low	Low	Low	Moderate risk of bias
Johns et al. (13)	Moderate	Low	Low	Low	Low	Low	Low	Moderate risk of bias
Kalem and SAhIN (25)	Serious	Low	Low	Low	Moderate	Low	Moderate	High risk of bias
Faour et al. (21)	Moderate	Low	Low	Low	Low	Low	Low	Moderate risk of bias
Johnson et al. (24)	Moderate	Low	Low	Low	Serious	Low	Low	High risk of bias
Sabater-Martos et al. (27)	Moderate	Low	Low	Low	Low	Low	Low	Moderate risk of bias
Kerbel et al. (26)	Moderate	Low	Low	Low	Low	Low	Low	Moderate risk of bias

### Recurrence of Infection

**Figure 2:**
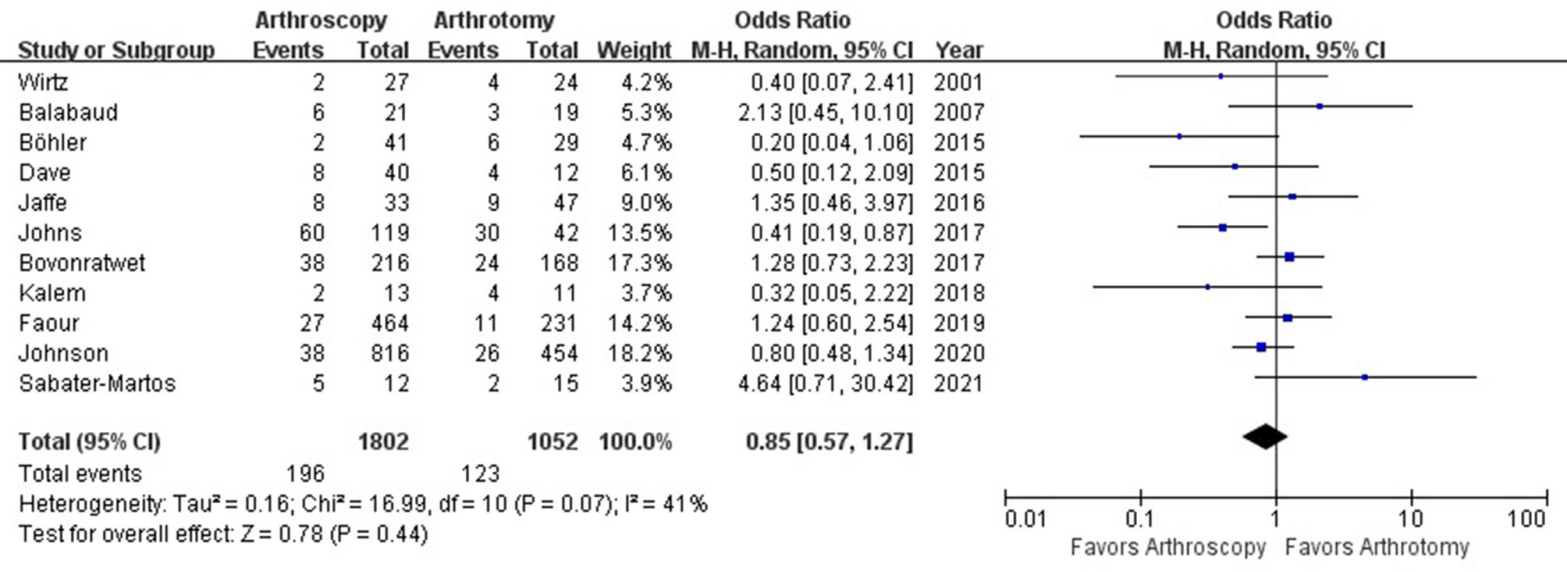
Forest plots of the comparison of arthroscopy and arthrotomy for reinfection rate.

**Figure 3:**
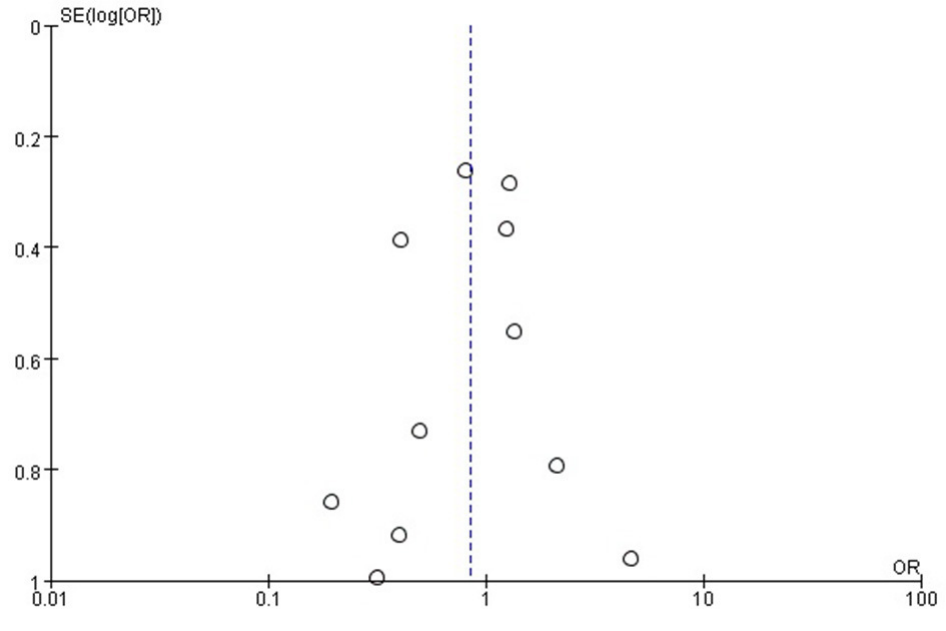
Funnel plots for detecting publication bias in the primary outcome of reinfection rate.

The rate of recurrence of infection were 10.9% (196 out of 1,802) and 11.7% (123 out of 1,052) in arthroscopy and arthrotomy group, respectively. Arthroscopy and arthrotomy management of the knee septic arthritis showed comparable rate of reinfection (OR = 0.85; 95% CI, 0.57–1.27; *P* = 0.44). This is shown in Figure 2. No significant heterogeneity was observed across studies (*I*^2^ = 41%). With no significant asymmetry was detected in the funnel plot, there was no evidence of publication bias in regard to recurrence of infection (Figure 3).

### Secondary Outcomes

**Figure 4:**
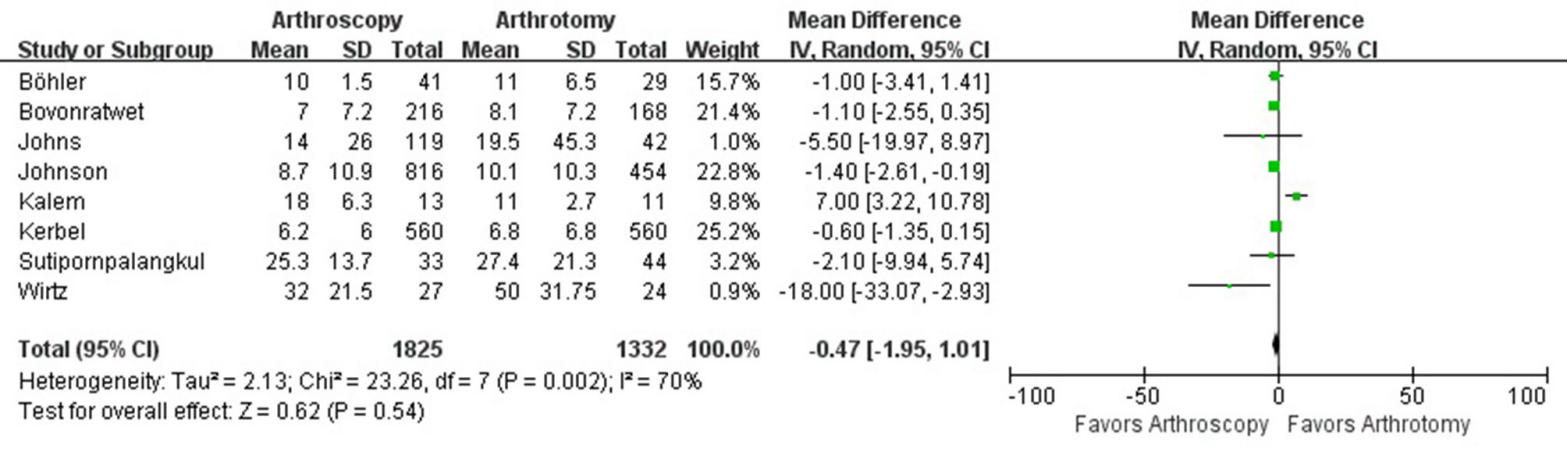
Forest plots of the comparison of arthroscopy and arthrotomy for hospital length of stay.

**Figure 5:**
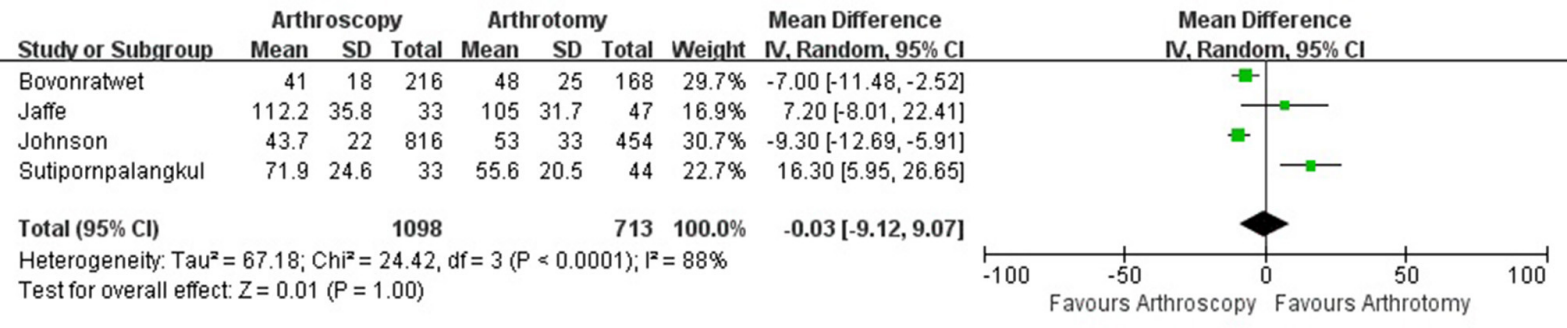
Forest plots of the comparison of arthroscopy and arthrotomy for operative time.

**Figure 6:**
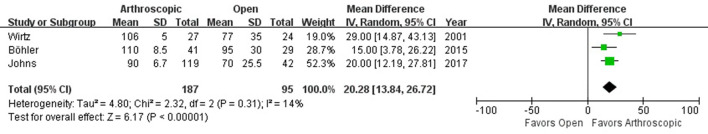
Forest plots of the comparison of arthroscopy and arthrotomy for postoperative range of motion.

**Figure 7:**
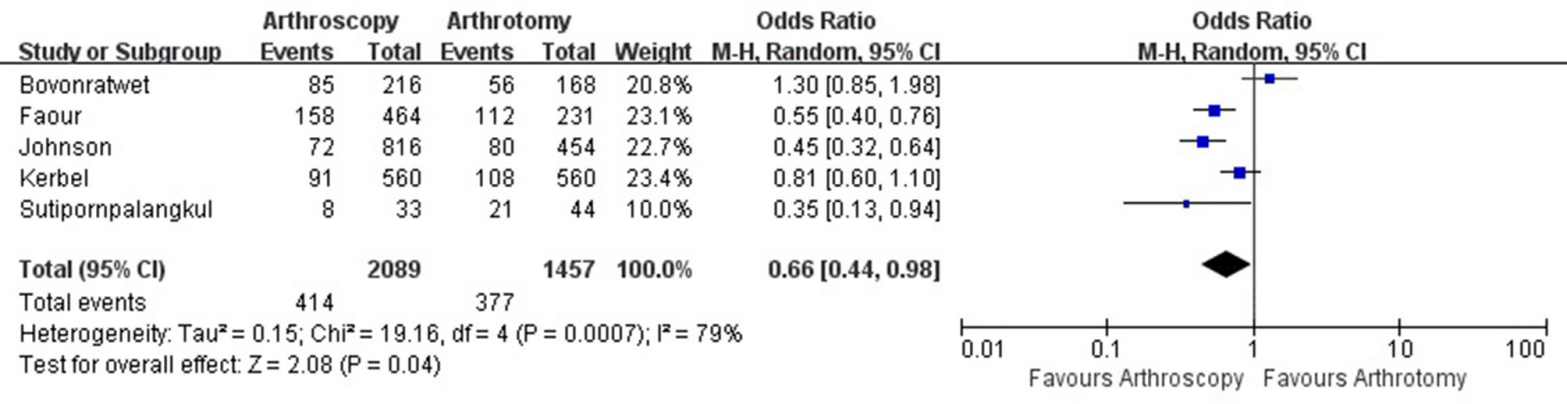
Forest plots of the comparison of arthroscopy and arthrotomy for overall complications.

**Figure 8:**
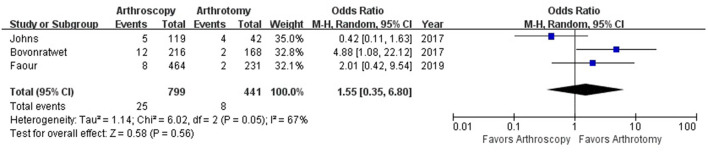
Forest plots of the comparison of arthroscopy and arthrotomy for mortality.

The hospital length of stay was reported in 8 studies with 1,825 knees treated with arthroscopy debridement and 1,332 treated with open debridement. No significant difference was observed in hospital length of stay between arthroscopy and arthrotomy management group (mean difference, −0.47; 95% CI, −1.95 to 1.01; *P* = 0.54; *I*^2^ = 70%) (Figure 4). Comparable operative time was found between arthroscopy and arthrotomy management (mean difference, −0.03; 95% CI, −9.12 to 9.07; *P* = 1.00; *I*^2^ = 88%) (Figure 5). The mean difference in postoperative range of motion was 20.28 degrees (95% CI, 13.84–26.72 degrees; *P* < 0.00001) in favor of arthroscopy management. No significant heterogeneity was observed across studies (*I*^2^ = 14%). This is shown in Figure 6. Arthroscopy management had significant lower rate of overall complication when compared to arthrotomy management (OR = 0.66; 95% CI, 0.44–0.98; *P* = 0.04; *I*^2^ = 79%; Figure 7). No significant difference was observed in mortality (OR = 1.55; 95% CI, 0.35–6.80; *P* = 0.56; *I*^2^ = 67%) between arthroscopy and arthrotomy management group (Figure 8). The significant heterogeneity found in hospital length of stay, operative time, overall complication rate and mortality may be attributed to clinical heterogeneity and explained by the fact that these events depend on the overall development of medical care and can be varied between different regions and hospitals.

## Discussion

Septic arthritis of the native knee is a serious condition which can be joint threatening and potentially life threatening. Immediate surgical debridement which can be performed either by arthroscopy or arthrotomy is essential to prevent devastating cartilage destruction and sepsis. To the best of our knowledge, there remains no consensus on which treatment is optimal for knee septic arthritis. The most important findings from this meta-analysis were that arthroscopy and arthrotomy showed similar efficacy in infection eradication in the treatment of native septic knee, and arthroscopy treatment was associated with better postoperative functional recovery and lower complication rate. Our results support the routinely application of arthroscopic debridement and irrigation for the management of the septic knee, while open debridement and irrigation can also be recommended when arthroscopic treatment is unavailable.

The efficacy of arthroscopy or arthrotomy in infection eradication in the treatment of septic knee has been debated for many years. While studies with controversial results have been published, the theoretical supports for arthroscopy or arthrotomy treatment have also been hypothesized by the authors. Surgeons favoring arthroscopy believed the reinfection rate was lower after arthroscopy management because arthroscopy is less invasive and causes less soft-tissue injury, which prevents spread of pathogen and infection (13, 29). Besides, arthroscopy provides a better access to both medial and lateral compartment as a whole and delivers a relatively closed joint when performing irrigation. As a result, the fluid can accumulate and irrigate the entire joint space more thoroughly, in contrast to the inadvertently escaped fluid in arthrotomy management. However, some surgeons believe open debridement and irrigation can have better operative exposure, and provide more adequate and definitive clearance of the joint space (14). The results of our study suggest both approaches have its advantages in infection eradication and can achieve comparable and satisfactory therapeutic efficiency.

It has been proved that minimally invasive procedure can have greater postoperative functional results when compared to arthrotomy procedure in many orthopedic conditions, such as repair of acute achilles tendon and repair of lateral ankle ligament (30, 31). We found significantly higher postoperative range of motion in arthroscopy group, and this is in accordance with the long-term superior functional results with arthroscopy management for knee septic arthritis of children (32). Peres et al. reported that the pain, warmth and redness of knee were significantly lower in the first week after surgery in knee septic arthritis patients treated with arthroscopy when compared to patients treated with arthrotomy (29), suggesting that less local inflammation and soft-tissue injury ensures rapid recovery after surgery and greater range of motion of knee.

Besides the better functional results identified in the arthroscopy group, we also found the complication rate associated with arthroscopy was lower than arthrotomy. It has been widely accepted that applying minimally invasive technique guarantees the lower complication rate with decreased surgical stress and fluctuation of the comorbidities (30, 31). Postoperative complications, such as bleeding complications and wound complications, are significantly reduced. However, the hospital length of stay, which is a significant indicator for postoperative recovery (33), was comparable between arthroscopy and arthrotomy group. The reason for this phenomenon may be the prolonged use of antibiotics in hospital and relatively slow recovery of septic knee patients no matter what kind of treatment is applied.

Joint culture plays an important role in the diagnosis of septic arthritis and the selection of specific antibiotics, while it is also worthy to mention that the joint culture may influence the selection of arthroscopy or arthrotomy treatment. Jaffe et al. found that Methicillin-Resistant Staphylococcus aureus (MRSA) infection was an independent risk factor for failure of a single surgical procedure in knee septic arthritis (11). Moreover, the arthroscopy management had a higher reinfection rate in treating MRAS infection of knee than arthrotomy treatment. Arthrotomy procedure with a thorough synovectomy was recommended by the author when managing MRAS infection. The culture-negative infection in knee septic arthritis was quite common with the reported rate ranging from 16.9 to 52.3% (11, 13, 29). Culture-negative infection was identified as a protective factor for reinfection, which may be explained by lower bacteria load (13). Paz and his colleagues also concluded patients with culture-negative native joint septic arthritis have less severe disease and better treatment outcomes in their study (34). However, whether septic knee with negative joint culture should specifically receive arthroscopy or arthrotomy to achieve better infection eradication efficacy remains unknown. Since septic knee with an early onset of the inflammatory symptoms may benefit more from an arthroscopic procedure, it is speculated that arthroscopy can be recommended for culture-negative septic arthritis (20). With limited information in the included studies, further subgroup analysis cannot be performed and definitive recommendation cannot be given in our study.

There are several limitations in this article. Firstly, only retrospective comparative studies were included in this systematic review. Given the fact that knee septic arthritis is an uncommon diagnosis, randomized controlled trials (RCT) in this field is often difficult to conduct. As a result, we included retrospective studies to determine the better treatment of choice. We believe a total of 4,051 septic arthritis knees gives it considerable power to detect the difference between these two procedures. Secondly, many variables which potentially affected the success rate of surgical management and postoperative knee function were unavailable from the included studies and further detailed subgroup analysis could not be performed for outcomes stratified by these variables. For example, joint culture, surgeon's technique, previous history of knee septic arthritis and preoperative knee range of motion may be confounding factors for estimating the infection eradication efficacy and postoperative knee function between arthroscopy and arthrotomy treatment (35). As a result, further high-quality studies with more details need to be conducted to draw a more comprehensive and detailed conclusion.

## Conclusion

Based on the current evidence, arthroscopy and arthrotomy had similar efficacy in infection eradication in the treatment of native septic knee. Nevertheless, arthroscopy treatment was associated with better postoperative functional recovery and lower complication rate. Our results support the routinely application of arthroscopic debridement and irrigation for the management of the septic knee, while open debridement and irrigation can also be recommended when arthroscopic treatment is unavailable. Additional high-quality trials are required to strengthen the evidence.

## Data Availability Statement

The original contributions presented in the study are included in the article/supplementary material, further inquiries can be directed to the corresponding author/s.

## Author Contributions

LL and JW: wrote protocol. ZL, XD, LL, and JW: data entry, checking, recoding, analysis, commented on and revised the manuscript draft for critical content, and approved the final version. XD: statistical guidance. ZL and LL: interpretation of statistical findings. ZL: wrote first draft of manuscript. All authors contributed to the article and approved the submitted version.

## Funding

This work was supported by the Key Science and Technology Project of Sichuan Province (Fund No. 2020YFS0153).

## Conflict of Interest

The authors declare that the research was conducted in the absence of any commercial or financial relationships that could be construed as a potential conflict of interest.

## Publisher's Note

All claims expressed in this article are solely those of the authors and do not necessarily represent those of their affiliated organizations, or those of the publisher, the editors and the reviewers. Any product that may be evaluated in this article, or claim that may be made by its manufacturer, is not guaranteed or endorsed by the publisher.
